# Investigation of the trend and related factors of suicide attempts and suicide deaths in southeast Iran during 2018–2022

**DOI:** 10.1038/s41598-024-63079-8

**Published:** 2024-05-29

**Authors:** Mohsen Rezaeian, Zahra Jamali, Zahra Javadi, Alireza Taherifard, Parvin Khalili

**Affiliations:** 1 Department of Epidemiology and Biostatistics, School of Health, Occupational Environment Research Center, Rafsanjan University of Medical Sciences, Rafsanjan, Iran; 2 Non-Communicable Diseases Research Center, Rafsanjan University of Medical Sciences, Rafsanjan, Iran; 3 Clinical Research Development Unit (CRDU), Niknafs Hospital, Rafsanjan University of Medical Sciences, Rafsanjan, Iran; 4 Social Determinants of Health Research Center, Rafsanjan University of Medical Sciences, Rafsanjan, Iran; 5 Department of Epidemiology, School of Public Health, Rafsanjan University of Medical Sciences, Rafsanjan, Iran

**Keywords:** Trend, Suicide, Suicide attempt, Completed suicide, Iran, Rafsanjan, Health care, Medical research

## Abstract

Suicide is a major public health challenge worldwide with an increasing trend. Identifying risk factors for suicide attempts and suicide deaths may help find useful ways to prevent suicide. We aimed to determine the trend and related factors of suicide attempts and suicide deaths in Rafsanjan. This retrospective study included all suicide cases from 2018 to 2022 in Rafsanjan, a city in the southeast of Iran. The information was extracted from the suicide registration system of Rafsanjan University of Medical Sciences. Univariable and multivariable logistics regression models were used to investigate factors affecting suicide death. A total of 2039 cases of suicide, including 1932 cases (94.75%) of suicide attempts and 107 cases (5.25%) of suicide deaths were recorded during the study period. The frequency of suicide deaths and suicide attempts per 100,000 people increased in 2022 compared to 2018 in both genders. In the adjusted model, the odds of suicide deaths in males was 6.48 (95% CI 3.39–12.42) times higher than in females. Also, the odds of suicide deaths in unemployed subjects and housewives were 2.64 (95% CI 1.50–4.67) and 7.45 (95% CI 3.08–18.07) times higher than employed subjects respectively. Finally, people with education less than a diploma had 10.85 (95% CI 1.48–79.54) times higher odds of suicide deaths compared to people with university education. The present research showed that the pattern of suicide has been increasing since 2018, and we may see an upward trend in the coming years, which requires further investigation and preventive measures. Male gender, low education level, unemployment, and being a housewife were associated with the highest frequency of suicide death.

## Introduction

Suicide is one of the main causes of unnatural death in the world^1^. Suicide is defined by World Health Organization (WHO), as an act in which a person hurts himself/herself consciously and without the intervention of others^2^. The increased incidence of suicides in recent years has attracted widespread national and international attention^2^. Due to this alarming growth, WHO and the international association for suicide prevention (IASP), have designated September 10 as World Suicide Prevention Da^3^.

WHO estimates that 703,000 individuals die by suicide annually, and suicide attempts are more than this statistic. Each suicide is a tragedy that affects families, communities, and nations, and leaves enduring impacts on survivors. Suicide occurs throughout life. It ranked as the fourth leading cause of death among individuals aged 15–29 worldwide in 2019^4^. Suicide does not happen only in high-income nations, but is a global phenomenon that transcends geographical boundaries. The majority of global suicides, exceeding 77%, occur in low- and middle-income countries^4^. Regional disparities in suicide rate are evident, with Scandinavian, German, Eastern European, Australian, and Japanese regions reporting higher suicide rates (25 cases per 100,000) compared to countries such as Spain, Italy, Ireland, the Netherlands, and Egypt (10 suicide cases per 100,000)^5^. Almost 60% of suicide cases occur in Asian countries. It seems that suicide has transitioned from Western Europe to Eastern Europe and nowadays, Asia has become the main place of the problem^6^. Notably, developing nations, particularly in the Eastern Mediterranean region, have witnessed a concerning uptick in suicide rates during the past decades^7^. Despite Iran’s comparatively lower suicide rate of 6.8% globally, recent studies underscore a worrisome trend of a 60% surge in the country’s suicide rate between 2015 and 2019, with an annual increase of 15%^2^. Comparing suicide rates in the second quarter of 2020 to the same period in the previous year reveals a notable surge in both the frequency and characteristics of suicide attempts^2^. According to the suicide studies, numerous factors are involved in suicide attempts^8–10^. In this regard, WHO refers to psychological and neurological factors^8^. Gender, education, and place of residence are other important causes of suicide^9^. Research has shown that the highest number of suicide attempts was in the age group of 15–25 years^11^. According to the studies conducted in Rafsanjan, there were 347 suicide attempts in 2015, three of which (0.9%) resulted in death^12^. Also, there were 339 suicide attempts in 2018, six of which (1.8%) resulted in death^13^. Given the escalating suicide rates in Iran^14^, and the impact of cultural and regional factors on suicide attempts^9,10, 15, 16^, this study aimed to investigate the trend and related factors of suicide attempts and suicide deaths in Rafsanjan during 2018–2022.

## Materials and methods

This retrospective study included all suicide cases from 2018 to 2022 in Rafsanjan, a city in the southeast of Iran. According to the statistics of 2023, 402,985 people, including 196,043 females and 206,942 males are covered by Rafsanjan University of Medical Sciences. This university covering the two cities of Rafsanjan and Anar, has three public hospitals: Ali Ibn Abi Talib, Moradi, and Valiasr.

There is a coherent system of information collection in Iran. Wherever a suicide attempt or suicide death occurs, its information is recorded and collected in this system. The registered information in this system includes information related to all suicide cases that were referred to the emergency departments of these three public hospitals and also includes information related to people who attempt suicide but do not go to the hospital due to death, but their information is registered in the Department of Forensic Medicine. The collected information is reported monthly by the emergency departments of hospitals and the Department of Forensic Medicine to the mental health department of the Health Deputy using a suicide prevention plan checklist. This checklist had two general parts. The first part included demographic information such as age, gender, education, employment, and marital status, and the second part included information related to suicide including history of suicide attempts, as well as the method of suicide and outcome of suicide (death or survival). Due to the non-availability of information related to a history of suicide attempts in some years, this variable was not included in the present study.

### Ethics approval and consent to participate

The present study was approved by the Ethics Committee of Rafsanjan University of Medical Sciences (Ethical Code ID: IR.RUMS.REC.1402.105). Written informed consent was obtained from the participants or their legal guardians. The data of Participants was kept confidential and was only accessible to the study investigators. All methods were performed according to the relevant guidelines and regulations.

### Statistical analyses

The statistical analysis of the data was conducted using Stata version 14 software. Descriptive statistics were used to present qualitative variables as frequencies and percentages, and quantitative variables as means and standard deviations. The chi-square test was employed for analyzing qualitative variables, while the independent t-test was utilized for quantitative variables. Binary logistic regression analysis was utilized to explore the factors influencing suicide deaths. The strength of the relationship between suicide deaths and relevant factors was assessed using odds ratios (ORs) and confidence intervals (CIs). Univariable and multivariable logistic regression analyses were performed to estimate ORs with 95% CIs. Initially, separate bivariate models were developed to determine the odds ratios with 95% confidence intervals for variables associated with suicide deaths, including gender (female and male), residency (city, village), age (years), education (illiterate, high school, diploma, university), marital status (single, married), and employment status (employed, unemployed, housewife, student/soldier). Subsequently, these variables were included in a multivariable logistic regression model to calculate adjusted ORs and 95% CIs for variables associated with suicide deaths while controlling for the influence of other variables. A significance level of P < 0.05 was considered for statistical analyses.

## Results

The results of this study showed that a total of 2039 cases of suicide, including 1932 cases (94.75%) of suicide attempts and 107 cases (5.25%) of suicide deaths were referred to the emergency departments of three public hospitals in Rafsanjan or their information was recorded in the Department of Forensic Medicine during 2018–2022.

As shown in Table 1, the frequency of suicide attempts and suicide deaths has been increasing from 2018 to 2022 in both genders. The highest frequency of suicide attempts and suicide deaths was related to 2022.

**Table1:** Frequency of suicide attempts and suicide deaths in Rafsanjan during 2018–2022 stratified by gender.

Year		2018	2019	2020	2021	2022
Suicide attempts.n (%)	Male	107 (14.38)	108 (14.52)	172 (23.12)	153 (20.56)	204 (27.42)
	Female	156 (13.27)	173 (14.71)	242 (20.58)	282 (23.98)	323 (27.47)
Suicide deaths. n (%)	Male	13 (18.31)	12 (16.90)	11 (15.49)	12 (16.90)	23 (32.39)
	Female	3(8.33)	4 (11.11)	8 (22.22)	10 (27.78)	11 (30.56)
Total. n (%)	Male	120 (14.72)	120 (14.72)	183 (22.45)	165 (20.25)	227 (27.85)
	Female	159 (13.12)	177 (14.60)	250 (20.63)	292 (24.09)	334 (27.56)

**Figure 1:**
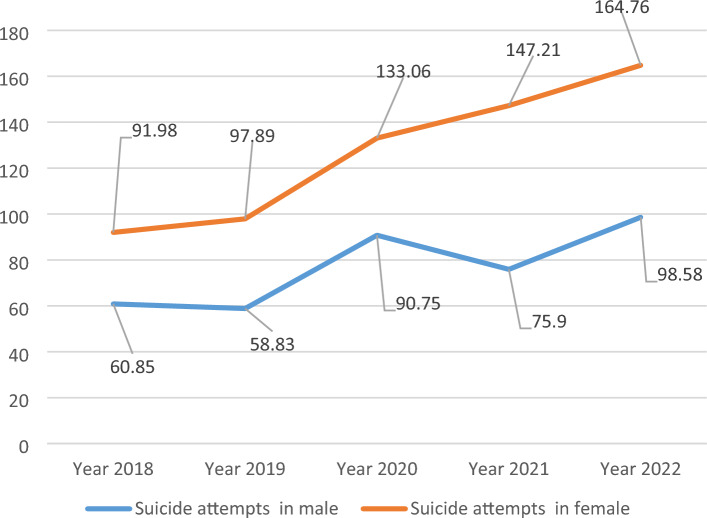
The 5 year trend of suicide attempts per 100,000 people in Rafsanjan during 2018–2022.

**Figure 2:**
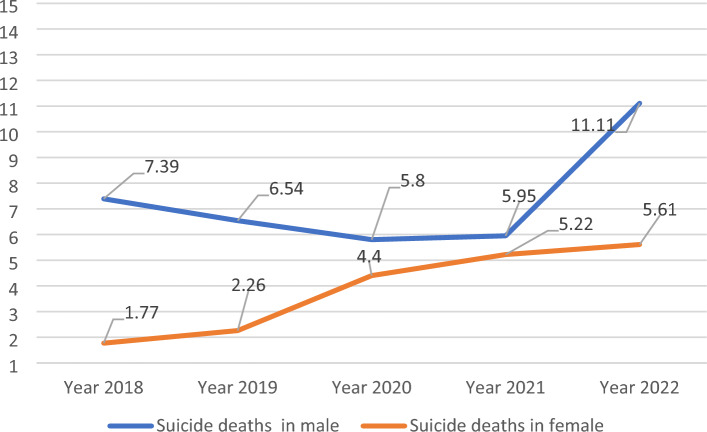
The 5 year trend of suicide death per 100,000 people in Rafsanjan during 2018–2022.

Figure 1 shows the 5-year trend of suicide deaths per 100,000 people in Rafsanjan. Figure 2 shows the 5-year trend of suicide attempts per 100,000 people in Rafsanjan. The frequency of suicide deaths and suicide attempts per 100,000 people increased in 2022 compared to 2018 in both genders.

Table 2 shows the frequency distribution of demographic characteristics according to suicide attempts and suicide deaths. The frequency of suicide attempts (61.25%) in females was significantly higher than males (38.75%), while suicide deaths in males (66.36%) were significantly higher than females (33.64%) (p < 0.001). The mean age of suicide cases in the present study was 26.12 ± 10.11 years, which was significantly higher in subjects who died after suicide (29.80 ± 11.94) than those who survived (25.92 ± 9.96) (p < 0.001). Also, most cases of suicide attempts were related to the age group of 19–24 years (26.60%) and the least cases were related to the age group of more than 32 years (21.89%). However, the frequency of suicide deaths was higher in people over 32 years old (36.45%).

**Table 2:** Frequency distribution of demographic characteristic according to suicide attempts and suicide deaths.

Variables	Total (n = 2039)	Suicide attempts (n = 1932)	Suicide deaths (n = 107)	P-value
Gender. n (%)				< 0.001
Male	815 (40.21)	744 (38.75)	71 (66.36)	
Female	1212 (59.79)	1176 (61.25)	36 (33.64)	
Unknown	12	12	0	
Age. n (%)				< 0.001
< 19	526 (25.80)	507 (26.24)	19 (17.76)	
19–24	541 (26.53)	514 (26.60)	27 (25.23)	
25–32	510 (25.01)	488 (25.26)	22 (20.56)	
≥ 32	462 (22.66)	423 (21.89)	39 (36.45)	
Mean ± SD	26.12 ± 10.11	9.96 ± 25.92	29.80 ± 11.94	0.004
Residency. n (%)				0.860
Urban	1326 (67.45)	1,253 (67.40)	73(68.22)	
Village	640 (32.55)	606 (32.60)	34(31.78)	
Unknown	73	73	0	
Education. n (%)				0.003
Illiterate	87 (4.37)	83 (4.40)	4 (3.81)	
High school	1150 (57.79)	1076 (57.08)	74 (70.48)	
Diploma	520 (26.13)	494 (26.21)	26 (24.76)	
University	233 (11.71)	232 (12.31)	1 (0.95)	
Unknown	49	47	2	
Marital status. n (%)				0.024
Single	964 (48.98)	929 (48.72)	50 (47.17)	
Married	861 (43.75)	837 (43.89)	55 (51.89)	
Divorced	143 (7.27)	141 (7.39)	1 (0.94)	
Unknown	26	25	1	
Employment. n (%)				0.142
Unemployed*	725 (35.56)	666 (35.94)	59 (55.66)	
Housewife	342 (16.77)	319 (17.22)	23 (21.70)	
Student	450 (22.07)	444 (23.96)	6 (5.66)	
Soldier	19 (0.93)	19 (1.03)	0 (0.00)	
Employed	393 (19.27)	375 (20.24)	18 (16.98)	
Others	30 (1.47)	30 (1.62)	0 (0.00)	
Unknown	80	79	1	
Suicide method. n (%)				< 0.001
Hanging	46 (2.26)	7 (0.36)	39 (36.45)	
Taking medical drugs	1551 (76.14)	1532 (79.38)	19 (17.76)	
Poisoning	209 (10.26)	171 (8.86)	38 (35.51)	
Substance use	164 (8.05)	162 (8.39)	2 (1.87)	
Others	67 (3.29)	58 (3.01)	9 (8.41)	
Unknown	2	2	0	

Unemployed*: It means both unemployed males and females who are not housewives.

Also, the frequency of suicide attempts (57.08%) and suicide deaths (70.48%) was higher in people with education less than a diploma (p = 0.003). Although, the frequency of suicide deaths in married people (51.89%) was significantly more than that of single people (47.17%), suicide attempts in single people (48.72%) were higher than that of married people (43.89%) (P = 0.024). In this study, there was no statistically significant difference between the frequency of suicide attempts and suicide deaths according to residency (P = 0.860) and also employment (p = 0.142) (Table 2).

There was a significant relationship between the method of suicide and the outcome of suicide (p < 0.001). The results of the research indicated that most suicide attempts were with the method of overdose of medical drugs (76.14%), but the frequency of suicide deaths was higher in other methods such as hanging (36.45%) and using agricultural poisons (35.51%) (Table 2).

Factors affecting suicide deaths based on logistic regression models are presented in Table 3. In univariable analysis, gender, age, education, and employment were related to suicide deaths. After controlling other factors, the results of multivariable analysis showed that the odds of suicide deaths in males was 6.48 (95% CI 3.39–12.42) times higher than females. Also, the odds of suicide deaths in unemployed subjects and housewives were 2.64 (95% CI 1.50–4.67) and 7.45 (95% CI 3.08–18.07) times higher than employed subjects respectively. Finally, people with education less than a diploma had 10.85 (95% CI 1.48–79.54) times higher odds of suicide deaths compared to people with a university education.

**Table 3:** Factors affecting suicide deaths based on logistic regression model.

Variables		Crude odds ratio confidence interval 95%	P-value	Adjusted odds ratio (95% confidence interval)	P-value
Gender	Female	1	–	1	–
	Man	**3.12 (2.07–4.70)**	** < 0.001**	**6.48 (3.39–12.42)**	** < 0.001**
Age. year*		**1.03 (1.02–1.05)**	** < 0.001**	1.015 (1.04-.99)	0.174
Residency	City	1	–	1	–
	Village	0.96(0.63–1.46)	0.860	0.903(0.58–1.41)	0.652
Education	University	1	–	1	–
	Illiterate	**11.18 (1.23–101.47)**	**0.032**	5.36 (0.57–49.97)	0.141
	High school	**15.96 (2.21 -115.36)**	**0.006**	**10.85 (1.48–79.54)**	**0.019**
	Diploma	**12.21 (1.65–90.53)**	**0.014**	6.76(0.90–50.84)	0.063
Marital status	Single	1	–	1	–
	Married	1.43(0.97–2.12)	0.073	1.19 (0.72–1.95)	0.504
Employment	Employed	1	–	1	–
	Unemployed*	**1.99 (1.16–3.43)**	**0.013**	**2.64 (1.50–4.67)**	**0.001**
	Housewife	1.62 (0.86–3.06)	0.135	**7.45 (3.08–18.07)**	** < 0.001**
	Student/soldier	**0.29 (0.74–0.11)**	**0.010**	0.71 (0.26–1.97)	0.514

*Age. Year (continuous): the odds of suicide deaths was calculated for each year of increase in the subject's age. Unemployed*: It means both unemployed males and females who are not housewives.

Significants values are in bold.

## Discussion

The results of the present study showed that approximately 2039 cases of suicide deaths and suicide attempts were recorded in Rafsanjan during 2018–2022. The trend of suicide attempts and suicide deaths increased with a gentle slope during these 5 years. This finding was consistent with the study of Afroozi et al. in the north of Iran that predicted the rate of suicide would increase in the next years based on modeling analysis^9^. Similarly, in the study of Razai et al., the rate of suicide showed an increasing trend in Iranian elderly^17^. In a systematic review and meta-analysis of articles published between 2010 and 2021 in Iran, although Iran was ranked with a low prevalence of suicide attempts and suicide deaths compared to the world average, however, the trend of suicide attempts was increasing and mostly affecting young people^2^. Unlike our study, the results of this systematic review study showed that the trend of suicide deaths was decreasing^2^. One of the possible reasons for this discrepancy can be due to the difference in the way of recording suicide data in different regions and also at different times.

According to the study of Alinejad et al.^13^ in, there were 339 suicide attempts in Rafsanjan, 6 of which (1.8%) resulted in death^13^. In the present study in 2018, there were 279 cases of suicide attempts, 16 of which (5.39%) resulted in death, which is a big difference in this regard. This difference could be due to the non-inclusion of registered cases of suicide death in the Department of Forensic Medicine in the study of Alinejad^13^, while in the present study, these registered cases of suicide death were also included. Furthermore, different cases of suicide attempts in the same year were observed in these two studies, which indicates the lack of accurate recording of suicide information. Therefore, a comprehensive investigation of the cause of this issue is very important. Since suicide is a public health problem, recording suicide attempts and suicide deaths will be used to facilitate understanding of basic epidemiological characteristics, monitoring its trends over time, assessing risk factors, comorbidities, and economic burden related to treatment, development of new treatment protocols as well as health care policies and ultimately improving people's quality of life. Registering suicide attempts and suicide deaths also help researchers have a data bank to conduct research studies in this field. However, there is a suicide registration system in Iran, this system has many flaws. Some cases of suicide may not be registered due to the lack of continuous monitoring of this system, and many important factors related to suicide, such as the history of mental disorders, socio-economic status, etc., are not registered in this system. Also, some of the recorded information has missing data such as the history of suicide attempts. Therefore, it is recommended to create a systematic program to record all the cases of suicide attempts and suicide deaths and also all related important factors.

Also, the present study showed that after controlling the effect of possible confounding factors in the adjusted model, gender (male), employment (unemployed, housewife), and education (below diploma) were related to suicide deaths. The frequency of suicide attempts was higher in females than males, but the frequency of suicide deaths was significantly higher among males. Also, in the adjusted model, the odds of suicide deaths in males was 6.48 (95% CI 3.39–12.42) times higher than females. This result was in agreement with some previous studies conducted in Iran by Mobasheri et al. in Chaharamahal and Bakhtiari^18^, Gorgi et al. in Shiraz^19^, Dadpour et al. in Mashhad^20^, and Afroozi et al. in Babol^9^ Moravveji et al. in Kashan^21^. It appears that males attempt suicide more seriously and employ more violent methods compared to females. The higher incidence of suicide attempts among females may be attributed to factors such as economic dependence, lack of social support, emotional issues, low self-esteem, and the oppressive nature of patriarchal structures within the family. Inconsistent with the results of the present study, research conducted by Shao et al. in China between 2003 and 2013 reported higher suicide deaths among females than males^22^. Also, in some previous studies^5,23^, suicide attempts were higher in males than females. This discrepancy could be attributed to variations in cultural and economic contexts across different regions globally^9^.

According to the results of univariable analysis in the present study, suicide attempts were more common in people under 24 years old, while suicide deaths were significantly higher in older people (over 32 years old). This finding was consistent with the results of studies conducted by Mirahmadizadeh et al. in Fars^24^ and Khademi et al. in Kermanshah^25^ that reported suicide attempts were more common in people under 24 years old. It was also consistent with the study of Afroozi et al. in Babol, who showed that although suicide attempts were higher among people under 40 years old, suicide deaths were higher among people over 40 years old^9^.

In the present study, although a statistically significant relationship was observed between age and suicide deaths based on the univariable results, this relationship was not observed in the results of the multivariable analysis after controlling the effect of confounding factors which was consistent with some previous studies^9,26^. Inconsistent with the result of the present study, in the study of Delam et al. conducted in Shiraz, increasing age was a predictor for suicide deaths after controlling the effect of confounder^27^. Also in a study by Grover et al. older age was associated with increased odds of suicide^28^.

Our study showed that in the adjusted model, the odds of suicide deaths in unemployed subjects and housewives were 2.64 (95% CI 1.50–4.67) and 7.45 (95% CI 3.08–18.07) times higher than employed subjects respectively. Similarly, in previous studies, there was a significant relationship between unemployment and suicide deaths^29,30^. This finding showed that unemployment played an important role in increasing the vulnerability of people, especially young people who have the highest frequency of suicide. Unemployed people and housewives may attempt suicide more because of despair about their future employment and this requires effective planning in this field.

Our findings also showed that in the adjusted model, people with education less than a diploma had 10.85 (95% CI 1.48–79.54) times higher odds of suicide deaths compared to people with university education. This finding was consistent with a previous study in Iran^31^. Also, in a systematic review of 33 meta-analyses published between 2008 and 2021, the risk of suicide deaths increased twofold in people with low education^29^. The higher frequency of suicide attempts and suicide deaths among people with lower levels of education may be due to their limited knowledge about the complications and consequences of suicide.

## Limitations

The most important strengths of this study included access to the data without using any sampling and providing information, resulting from a large number of samples from different sources in a wide geographic range that also showed the trend during 5 years. However, this study has limitations such as the possibility of low reporting in suicide data (due to social stigma and legal issues), and the possible influence of other variables that may affect the assessment of causal relationships on suicide. In this study, factors affecting suicide such as social indicators (job status, socio-economic conditions, drug abuse, and alcohol consumption), and history of mental disorders in suicide cases were not available. Therefore, further research focusing on these factors and the characteristics of people with a positive history of suicide attempts is recommended. Also, some variables had missing data, which can affect the obtained results. It is therefore recommended to implement a systematic program for documenting the characteristics of individuals who attempt suicide for comprehensive and practical investigations. Furthermore, it is advisable to conduct further research on variables associated with suicide to accurately and scientifically identify the factors influencing suicide attempts. This will enable healthcare professionals and social institutions to develop targeted interventions and preventative measures.

## Conclusion

The present research showed that the pattern of suicide has been increasing since 2018, and we may see an upward trend in the coming years, signaling the necessity for further investigation and implementation of preventive measures. Findings from this study indicated that while females exhibit a higher frequency of suicide attempts than males, males experience a higher frequency of suicide deaths, suggesting a more serious intent among male individuals. Addressing this gender disparity could prove instrumental in mitigating the prevalence of suicide in society. Additionally, individuals with lower levels of education, unemployment status, and housewives were identified as having the highest frequency of suicide deaths. Hence, it is recommended that health policymakers take proactive measures to promote employment opportunities for young individuals to prevent this social issue and the depletion of active labor within the community. Strategic planning should also encompass the enhancement of advisory, educational, welfare, and social services to address the underlying factors contributing to suicide among vulnerable populations. Overall, the findings of the present study can help us find useful ways to prevent suicide and provide valuable information for the needs assessment, design, and revision of health and development programs of the country.

## Data Availability

Data sharing requests should be directed to Mohsen Rezaeian (moeygmr2@yahoo.co.uk) or Parvin Khalili (parvinkhalili61@yahoo.com).
